# Gender-Specific Associations of Muscle Strength With Diabetic Retinopathy in Middle-Aged and Older Chinese Adults: A Cross-Sectional Study

**DOI:** 10.1155/ije/8219457

**Published:** 2025-11-27

**Authors:** Jin-Jin Gu, Shan-Hu Qiu, Ying Xu, Yu-Zhi Ding, Xiao-Ying Zhou, Yan Liu, Yang Yuan, Zi-Lin Sun

**Affiliations:** ^1^Department of Endocrinology, Zhongda Hospital, Institute of Diabetes, School of Medicine, Southeast University, Nanjing, China; ^2^Department of Geriatrics, The Affiliated People's Hospital of Jiangsu University, Zhenjiang, China; ^3^Department of General Practice, Zhongda Hospital, Institute of Diabetes, School of Medicine, Southeast University, Nanjing, China; ^4^Department of Endocrine Metabolism, The First People's Hospital of Yunnan Province, The Affiliated Hospital of Kunming University of Science and Technology, Kunming, China; ^5^Department of Ophthalmology, Zhongda Hospital, Institute of Diabetes, School of Medicine, Southeast University, Nanjing, China

**Keywords:** chair stand test, cross-sectional study, diabetic retinopathy, gender differences, grip strength

## Abstract

**Background:**

Muscle weakness is associated with an increased risk of diabetes. However, its relationship with diabetic retinopathy (DR) in different genders remains unclear. This study aimed to investigate gender-specific associations between grip strength, lower limb function (assessed by the 30-s chair stand test, CST-30), and the prevalence of DR.

**Methods:**

We conducted a cross-sectional analysis of 962 adults with diabetes aged 45 years or older. Participants underwent grip strength testing, a CST-30 assessment, and retinal examinations graded according to the Early Treatment DR Study (ETDRS) classification. Low grip strength was defined as less than 28 kg in men and less than 18 kg in women. Participants in the lowest two gender-specific quintiles of CST-30 scores were categorized as having reduced lower limb strength. Pearson correlation analysis was used to assess the relationship between grip strength and CST-30, and logistic regression analysis was employed to evaluate the associations with DR.

**Results:**

Of the 962 participants with diabetes included, 404 were men. Grip strength was significantly but only modestly correlated with CST-30 in both men and women (*r* = 0.278 and 0.269, respectively). Further analysis revealed that, in men, low grip strength was independently associated with higher odds of DR (odds ratio [OR] = 2.98; 95% confidence interval [CI]: 1.33–6.68; *p*=0.008). However, reduced lower limb strength was not related to DR. In women, low grip strength was not associated with DR; however, reduced lower limb strength was significantly associated with DR (OR = 2.26; 95% CI: 1.18–4.34; *p*=0.014).

**Conclusion:**

Our findings suggest that in middle-aged and older Chinese adults with diabetes, DR was related to grip strength in men but to lower limb strength in women, indicating a gender-specific difference.

## 1. Introduction

Diabetic retinopathy (DR) is one of the most common microvascular complications of diabetes mellitus and the leading cause of vision loss worldwide [1–3]. In China, approximately 22% of people with diabetes have DR, and an estimated 7.7% develop DR annually [4, 5]. Studies have shown that poor glycemic control and hypertension are strongly associated with DR [6]. However, despite optimal glycemic and blood pressure (BP) control, DR still exists in some patients [7]. This indicates that additional factors may contribute to DR pathogenesis.

Muscle health is critical in the prevention of metabolic and vascular complications [8, 9]. Evidence suggests that skeletal muscle can influence glucose regulation and systemic inflammation by the release of a series of myokines (e.g., irisin) [10–13]. Muscle strength is a widely used indicator of muscle health, and prior studies have shown that reduced muscle strength is associated with structural retinal changes, including choroidal and retinal thinning [14]. However, few studies have examined the relationship between muscle strength and DR. A cross-sectional study in a Japanese population reported that low grip strength was related to proliferative DR [15]. Yet that study had a relatively small sample size (316 patients), and its predominantly outpatient sample may limit generalizability to community-dwelling individuals. Moreover, despite modest correlations between grip strength and the 30-s chair stand test (CST-30)—another measure of muscle strength [16, 17]—it is unclear whether CST-30 is associated with DR. In addition, gender differences in sarcopenia prevalence are well documented [18], making it clinically relevant to investigate whether gender modifies the association between muscle strength and DR.

Given these considerations, this study aimed to examine the association between muscle strength (assessed by grip strength and CST-30) and DR in middle-aged and older Chinese adults with diabetes, specifically focusing on gender differences by performing gender-stratified analyses.

## 2. Materials and Methods

### 2.1. Study Design and Participants

This cross-sectional study used data from the third survey wave of the SENSIBLE-Cohort, which enrolled 6310 community residents aged 18 years and older across eight provinces in China from April 2020 to January 2021 [19, 20]. The SENSIBLE-Cohort's study design and baseline survey procedures have been described previously [21].

**Figure 1:**
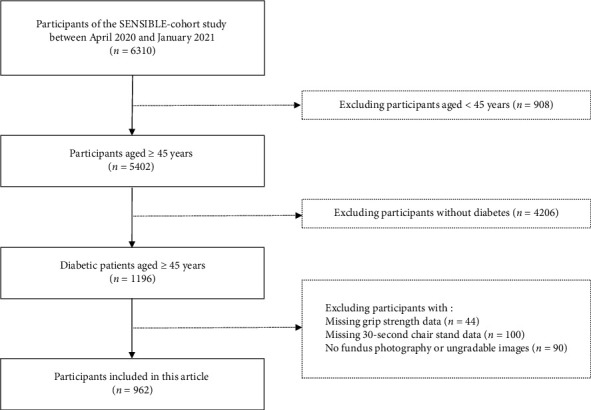
Flowchart showing participants' inclusion and exclusion criteria.

In this study, we included participants with diabetes who were aged ≥ 45 years and who underwent both fundus photography and muscle-strength assessment. Exclusion criteria were (1) age < 45 years; (2) nondiabetic individuals; (3) missing data on grip strength or the CST-30; (4) no fundus photographs or ungradable images; and (5) severe physiological or mental health conditions (e.g., dementia). Following these criteria, a total of 962 participants with diabetes were included in the analysis (Figure 1). The study was conducted and reported in accordance with the STROBE guidelines for observational studies [22].

### 2.2. Ethics Statement

The study protocol was approved by the Ethical Review Committee of Zhongda Hospital, Southeast University, Nanjing, China (Approval No. 2016ZDSYLL092-P01). Written informed consent was obtained from all participants before data collection, in accordance with the Declaration of Helsinki and relevant ethical standards.

### 2.3. Muscle Strength Assessment

#### 2.3.1. Grip Strength

Grip strength was measured using a calibrated digital hydraulic dynamometer (JAMAR, Patterson Medical, Cedarburg, WI, USA) following a standardized protocol [23]. Participants sat with their elbows flexed at 90° and were instructed to exert maximal grip force for at least 5 s. Two trials were performed with the dominant hand, and the maximum value was used for analysis. Based on established criteria for sarcopenia in the Chinese population, low grip strength was defined as < 28 kg for men and < 18 kg for women [18, 24].

#### 2.3.2. CST-30

The CST-30 was used to evaluate lower-extremity muscle strength and endurance [25]. Participants were asked to stand up and sit down from a chair as many times as possible within 30 s. The total number of sit-and-stands was recorded. Due to the absence of recommended cut-off points for low lower limb strength, participants were deemed to have low lower limb strength if their CST-30 score was within the lowest two gender-specific quintiles.

### 2.4. DR Assessment

Fundus photography was taken using a Canon nonmydriatic digital retinal camera (CR-2AF) to obtain standardized images centered on each eye's optic disc and macula. These images were digitally transmitted to Beijing Tongren Hospital, Capital Medical University, for centralized assessment. Two independent and masked retinal specialists graded the photographs utilizing the Early Treatment DR Study (ETDRS) classification system. The presence of DR was defined as an ETDRS level of 20 or higher in either eye [26].

### 2.5. Data Collection and Potential Covariates

Demographic characteristics, lifestyle factors, and medical history were collected by trained staff. Anthropometric measurements included height, weight, and body mass index (BMI). BP was measured after a five-minute rest using an automated sphygmomanometer (YE680E; Yuwell, Danyang, China) according to standardized protocols [21]. BMI was calculated as weight in kilograms divided by height in square meters. Smoking status was defined as having smoked at least 100 cigarettes in a lifetime [27]. Cardiovascular disease (CVD) was defined as a history of heart disease or stroke [28].

Fasting venous blood samples were collected after at least 10 h of fasting. For participants without known diabetes, an oral glucose tolerance test (OGTT) was performed. Fasting blood glucose (FBG), 2-h postprandial glucose (PBG), triglycerides, total cholesterol, high-density lipoprotein (HDL) cholesterol, and low-density lipoprotein (LDL) cholesterol were measured using an automated chemistry analyzer (Synchron LX-20; Beckman Coulter Inc., Carlsbad, CA, USA). Glycated hemoglobin A1c (HbA1c) was measured by high-performance liquid chromatography (D-10™ Hemoglobin Analyzer; Bio-Rad Laboratories, Hercules, CA, USA).

Diabetes was diagnosed according to American Diabetes Association (ADA) criteria: FBG ≥ 7.0 mmol/L; 2-h PBG ≥ 11.1 mmol/L; HbA1c ≥ 6.5%; a self-reported history of diabetes; or the use of antidiabetic medications [29]. Hypertension was defined as systolic BP ≥ 140 mmHg, diastolic BP ≥ 90 mmHg, a self-reported history, or the use of antihypertensive medications [30]. Hyperlipidemia was diagnosed according to National Cholesterol Education Program Adult Treatment Panel III (NCEP ATP III) criteria: total cholesterol ≥ 5.17 mmol/L, HDL-C < 1.04 mmol/L, triglycerides ≥ 1.70 mmol/L, LDL-C ≥ 3.37 mmol/L, or a history of hyperlipidemia [31].

### 2.6. Statistical Analysis

Descriptive statistics were used to summarize the baseline characteristics of the study population. Continuous variables are presented as means ± standard deviations (SD) for normally distributed data or as medians with interquartile ranges (IQR) for non-normally distributed data. Categorical variables are reported as frequencies and percentages. Group differences for continuous variables were assessed with Student's *t*-tests or Kruskal–Wallis tests, as appropriate, and categorical differences were evaluated with chi-square tests.

The relationship between grip strength and the CST-30 performance was assessed using Pearson correlation analysis. Associations between muscle strength and the odds of DR were estimated with logistic regression analyses performed separately for men and women, yielding odds ratios (ORs) and 95% confidence intervals (CIs).

For the analysis, several adjustment models were used: an unadjusted baseline model; Model 1 (adjusted for age and ethnicity); Model 2 (additionally adjusted for occupation, annual household income, smoking status, CVD, hypertension, hyperlipidemia, BMI, HbA1c, and diabetes duration); and Model 3 (further adjusted for low CST-30 performance or low grip strength, where appropriate). All the covariates were selected based on their clinical relevance with reference to the 2023 ADA guidelines and the statistical significance in univariate comparisons between groups [2]. Multicollinearity was assessed using variance inflation factors (VIFs), with a VIF < 5 indicative of no evidence of multicollinearity (Supporting Table 1).

Sensitivity analyses were conducted to assess the robustness of the primary findings. First, DR was redefined using an ETDRS level of 14 or higher [26, 32]. Second, missing data for occupation, HbA1c, and annual household income were imputed using multiple imputation. Third, grip strength and CST-30 were analyzed as continuous variables (per unit increase), and the shape of their associations with DR was evaluated using generalized additive models (GAMs) with spline smoothing.

All analyses were performed using R software (version 4.2.0) and EmpowerStats (X&Y Solutions, Inc., Boston, MA). A two-sided *p* value < 0.05 was considered statistically significant.

## 3. Results

### 3.1. Baseline Characteristics

Table 1 summarizes the demographic and clinical characteristics of the study population. A total of 962 participants were included, with a mean age of 59.05 years. There were 558 women and 404 men, and the prevalence of DR was slightly higher in men (12.87%) compared to women (11.65%).

Among women, there were no significant differences in age, ethnicity, occupation, annual household income, smoking status, hypertension, hyperlipidemia, BMI, or history of CVD between those with and without DR. However, women with DR exhibited significantly higher HbA1c levels (8.43% vs. 6.87%; *p* < 0.001). Grip strength did not differ significantly between groups, although there was a trend toward a higher prevalence of low CST-30 performance in women with DR (47.69% vs. 35.50%; *p*=0.055).

Among men, individuals with DR had significantly higher HbA1c levels (8.24% vs. 7.01%, *p* < 0.001) and longer diabetes duration (median 6.00 vs. 3.00 years, *p* < 0.001). Additionally, a higher proportion of men with DR had CVD (19.23% vs. 9.38%, *p*=0.031) and low grip strength (28.85% vs. 13.35%, *p*=0.004). No significant differences were observed in age, ethnicity, occupation, household income, smoking, hypertension, hyperlipidemia, or BMI across DR status in men.

### 3.2. Correlations Between Grip Strength and CST-30

**Figure 2:**
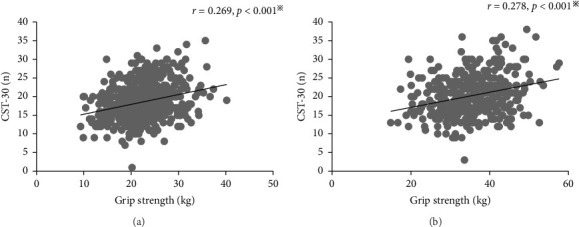
Relationships between grip strength and CST-30 with Pearson's correlation coefficient (*r*) values in the women (a) and men (b) populations. ^※^*p* < 0.05.

Pearson correlation analysis (Figure 2) shows that there were mild correlations between grip strength and CST-30 performance in both women (*r* = 0.269; *p* < 0.001) and men (*r* = 0.278; *p* < 0.001).

### 3.3. Univariate Associations of Covariates With DR

Table 2 shows the univariate associations between various covariates and DR. In women, higher HbA1c levels (OR = 1.75; 95% CI: 1.50–2.05; *p* < 0.001) and longer diabetes duration (OR = 1.14; 95% CI: 1.09–1.19; *p* < 0.001) were significantly associated with increased odds of DR. However, there were no significant associations with other covariates, such as age, occupation, ethnicity, and household income.

In men, higher HbA1c (OR = 1.58; 95% CI: 1.33–1.89; *p* < 0.001) and longer diabetes duration (OR = 1.15; 95% CI: 1.09–1.20; *p* < 0.001) also correlated with DR risk. Moreover, the Han ethnicity was associated with lower odds of DR than other ethnic groups (OR = 0.46; 95% CI: 0.23–0.89; *p*=0.021). However, the presence of CVD was associated with a higher DR risk (OR = 2.30; 95% CI: 1.06–5.01; *p*=0.036).

### 3.4. Multivariate Regression Analysis of Muscle Strength Indicators

Table 3 shows the multivariate logistic regression analyses between muscle strength and DR. In women, low CST-30 was significantly associated with increased odds of DR across all adjusted models. Specifically, low CST-30 was associated with an OR of 2.25 (95% CI: 1.18–4.32; *p*=0.014) in Model 2. This association remained significant in Model 3 (OR = 2.26, 95% CI: 1.18–4.34; *p*=0.014). In contrast, low grip strength was not associated with DR in women (all *p* > 0.05).

In men, low grip strength was consistently correlated with higher odds of DR. In Model 2, the OR was 2.96 (95% CI: 1.32–6.64; *p*=0.009), and the association remained significant in Model 3 (OR = 2.98; 95% CI: 1.33–6.68; *p*=0.008). In contrast, low CST-30 was not associated with DR (*p* > 0.05).

### 3.5. Sensitivity Analysis

Sensitivity analyses by redefining DR as an ETDRS level ≥ 14 (Supporting Table 2) or including participants with data imputation (Supporting Table 3) showed similar outcomes: low grip strength was related to the odds of DR in men but not in women. In contrast, low CST-30 was only associated with increased odds of DR in women. However, when grip strength and CST-30 were analyzed as continuous variables, their associations with DR became nonsignificant (Supporting Table 4), although GAMs indicated no evidence of nonlinearity for either gender (all *p* for nonlinearity > 0.2; Supporting Figure 1).

## 4. Discussion

This cross-sectional study revealed significant gender-specific associations between muscle strength and DR in middle-aged and older Chinese adults with diabetes. As summarized in Table 3, reduced grip strength was independently associated with higher DR prevalence in men, whereas poorer CST-30 performance (indicating reduced lower limb strength and function) was linked to DR in women. These findings suggest that upper- and lower-body muscle strength may reflect distinct pathophysiological pathways influencing microvascular health, with notable divergence between genders.

In line with a previous cross-sectional study of Japanese outpatients with type 2 diabetes, which reported an association between low grip strength and proliferative DR (OR = 6.25, 95% CI: 1.15–33.96) [15], our study showed that reduced muscle strength is also linked to higher odds of DR among community-dwelling adults with diabetes. By evaluating both upper- and lower-limb muscle strength via grip strength and CST-30, respectively, we identified a gender-specific difference in their association with DR (Table 3): low grip strength was associated with increased odds of DR in men but not in women, whereas poor CST-30 performance was associated with higher odds of DR in women but not in men.

The observed gender-specific associations may reflect divergent pathways of both muscle and metabolic aging between men and women. First, grip strength and CST-30 capture independent physiological attributes, as evidenced by their modest correlations (women: *r* = 0.269; men: *r* = 0.278; Figure 2). This low level of concordance is consistent with reports from large population-based cohorts [16]. Second, in men, the marked age-related decline in grip strength serves as a sensitive biomarker of systemic dynapenia, which is closely associated with decreasing testosterone levels and subsequent impairments in insulin sensitivity and microvascular integrity [33, 34]. Third, in women, the stronger association with CST-30 performance aligns with the rapid postmenopausal loss of dynamic lower-limb strength—a process often accompanied by increased visceral adiposity and worsening insulin resistance [35, 36]. Together, these findings suggest that gender-specific mechanisms of muscle dysfunction, rather than interchangeable strength measurements, contribute to the differences in the risk of DR.

Several mechanisms may explain the association between muscle strength and DR. First, reduced muscle strength reflects poorer physical function and lower activity levels, which can exacerbate metabolic dysregulation and impair glycemic control, promoting retinal microvascular damage [37]. Second, lower muscle strength is associated with disrupted myokine signaling and reduced systemic anti-inflammatory and antioxidant capacity, which may intensify vascular inflammation and oxidative stress related to DR [37, 38]. Third, reduced strength can contribute to insulin resistance and dyslipidemia [39, 40], which are established drivers of microvascular dysfunction, including DR [41].

The key strengths of this study include the use of validated muscle strength assessments and standardized, masked grading of DR according to the ETDRS protocol. The robustness of the primary findings is further supported by their consistency across multiple sensitivity analyses.

However, several limitations must be acknowledged. First, the cross-sectional design precludes causal inference. Second, the lack of a universally accepted threshold for defining low limb function using the CST-30 may limit cross-population comparability. Third, residual confounding by unmeasured factors (e.g., physical activity) may persist despite multivariable adjustment. Moreover, although individuals with known severe conditions were excluded, the absence of a systematic screening protocol for specific advanced organ diseases (e.g., cardiac, respiratory, hepatic, or renal) must be considered a potential source of residual confounding; that said, given the community-based setting and the relative rarity of such conditions, their overall impact is likely minimal. Fourth, although our sample size is considerable, statistical power may still have been insufficient to detect more subtle associations when muscle strength was analyzed as a continuous variable. Finally, the inability to distinguish between type 1 and type 2 diabetes due to a lack of data on C-peptide and islet autoantibodies should be noted. Nevertheless, given the low prevalence of type 1 diabetes in adults over 30 years of age (< 5%), our findings are likely generalizable to the majority of middle-aged and older adults with type 2 diabetes.

## 5. Conclusions

In summary, this study indicates that DR is associated with low grip strength in men and reduced lower limb strength in women among middle-aged and older Chinese adults with diabetes, highlighting a gender-specific difference. Our results support the use of these inexpensive and readily applicable tests in clinical practice to identify individuals at high risk for DR, thereby facilitating earlier retinal screening and targeted intervention. Future longitudinal studies are needed to establish causality between muscle strength and DR and to validate these gender-specific associations in other ethnically diverse populations.

## Figures and Tables

**Table 1:** Baseline characteristics of participants stratified by DR status and gender (*N* = 962).

Variables	Women (*n* = 558)		*p* value	Men (*n* = 404)		*p* value
	Non-DR (*n* = 493)	DR (*n* = 65)		Non-DR (*n* = 352)	DR (*n* = 52)	
Age, years	58.92 ± 6.55	57.94 ± 6.72	0.256	59.22 ± 7.15	59.17 ± 6.61	0.965
Ethnicity, *n* (%)			0.693			**0.019**
Han	426 (86.41%)	55 (84.62%)		297 (84.38%)	37 (71.15%)	
Other	67 (13.59%)	10 (15.38%)		55 (15.62%)	15 (28.85%)	
Occupation^∗^, *n* (%)			0.711			0.727
Workers	80 (16.29%)	8 (12.50%)		75 (21.31%)	13 (25.00%)	
Farmers	324 (65.99%)	45 (70.31%)		203 (57.67%)	27 (51.92%)	
Other	87 (17.72%)	11 (17.19%)		74 (21.02%)	12 (23.08%)	
Annual household income^∗^, *n* (%)			0.407			0.078
< 60,000, yuan	370 (75.51%)	46 (70.77%)		232 (66.10%)	40 (78.43%)	
≥ 60,000, yuan	120 (24.49%)	19 (29.23%)		119 (33.90%)	11 (21.57%)	
Smoking status, *n* (%)			0.434			0.325
No	479 (97.16%)	62 (95.38%)		161 (45.74%)	20 (38.46%)	
Yes	14 (2.84%)	3 (4.62%)		191 (54.26%)	32 (61.54%)	
CVD, *n* (%)			0.391			**0.031**
No	429 (87.02%)	59 (90.77%)		319 (90.62%)	42 (80.77%)	
Yes	64 (12.98%)	6 (9.23%)		33 (9.38%)	10 (19.23%)	
Hypertension			0.974			0.795
No	128 (25.96%)	17 (26.15%)		94 (26.70%)	13 (25.00%)	
Yes	365 (74.04%)	48 (73.85%)		258 (73.30%)	39 (75.00%)	
Hyperlipidemia			0.378			0.367
No	148 (30.02%)	23 (35.38%)		131 (37.22%)	16 (30.77%)	
Yes	345 (69.98%)	42 (64.62%)		221 (62.78%)	36 (69.23%)	
BMI, kg/m^2^	26.17 ± 3.87	25.51 ± 3.42	0.187	26.13 ± 3.57	26.46 ± 3.36	0.536
HbA1c^∗^, %	6.87 ± 1.23	8.43 ± 2.03	**<** **0.001**	7.01 ± 1.37	8.24 ± 1.68	**<** **0.001**
Duration of diabetes, years	3.00 (0–3.00)	3.00 (3.00–10.00)	**<** **0.001**	3.00 (0–3.00)	6.00 (3.00–10.20)	**<** **0.001**
Low grip strength, *n* (%)	81 (16.43%)	9 (13.85%)	0.594	47 (13.35%)	15 (28.85%)	**0.004**
Low CST-30, *n* (%)	175 (35.50%)	31 (47.69%)	0.055	121 (34.38%)	18 (34.62%)	0.973

*Note:* Bold *p* values represent < 0.05. Data are expressed as mean  ±  SD or median (Q1–Q3) for continuous variables, and as *n* (%) for categorical variables.

Abbreviations: BMI = body mass index; CST-30 = 30 s chair stand test; CVD = cardiovascular disease; DR = diabetic retinopathy; HbA1c = hemoglobin A1c.

^∗^Variables with missing data: occupation (*n* = 3), HbA1c (*n* = 4), annual household income (*n* = 5).

**Table 2:** Univariate analysis for DR.

Covariates	Women (*n* = 558)		*p* value	Men (*n* = 404)		*p* value
	Statistics	OR (95% CI)		Statistics	OR (95% CI)	
Age, years	58.81 ± 6.57	0.98 (0.94, 1.02)	0.256	59.21 ± 7.07	1.00 (0.96, 1.04)	0.965
Ethnicity, *n* (%)						
Other	77 (13.80%)	Reference		70 (17.33%)	Reference	
Han	481 (86.20%)	0.87 (0.42, 1.78)	0.694	334 (82.67%)	0.46 (0.23, 0.89)	**0.021**
Occupation, *n* (%)						
Workers	88 (15.86%)	Reference		88 (21.78%)	Reference	
Farmers	369 (66.49%)	1.39 (0.63, 3.06)	0.416	230 (56.93%)	0.77 (0.38, 1.56)	0.466
Other	98 (17.66%)	1.26 (0.48, 3.30)	0.632	86 (21.29%)	0.94 (0.40, 2.18)	0.878
Annual household income, *n* (%)						
< 60,000, yuan	416 (74.95%)	Reference		272 (67.66%)	Reference	
≥ 60,000, yuan	139 (25.05%)	1.27 (0.72, 2.26)	0.408	130 (32.34%)	0.54 (0.27, 1.08)	0.082
Smoking status, *n* (%)						
No	541 (96.95%)	Reference		181 (44.80%)	Reference	
Yes	17 (3.05%)	1.66 (0.46, 5.92)	0.438	223 (55.20%)	1.35 (0.74, 2.45)	0.326
CVD, *n* (%)			0.393			**0.036**
No	488 (87.46%)	Reference		361 (89.36%)	Reference	
Yes	70 (12.54%)	0.68 (0.28, 1.64)		43 (10.64%)	2.30 (1.06, 5.01)	
Hypertension			0.974			0.795
No	145 (25.99%)	Reference		107 (26.49%)	Reference	
Yes	413 (74.01%)	0.99 (0.55, 1.78)		297 (73.51%)	1.09 (0.56, 2.14)	
Hyperlipidemia			0.379			0.368
No	171 (30.65%)	Reference		147 (36.39%)	Reference	
Yes	387 (69.35%)	0.78 (0.45, 1.35)		257 (63.61%)	1.33 (0.71, 2.50)	
BMI, kg/m^2^	26.09 ± 3.82	0.95 (0.89, 1.02)	0.187	26.17 ± 3.54	1.03 (0.95, 1.11)	0.535
HbA1c, %	7.05 ± 1.43	1.75 (1.50, 2.05)	**<** **0.001**	7.17 ± 1.47	1.58 (1.33, 1.89)	**<** **0.001**
Duration of diabetes, *n* (%)	3.54 ± 4.48	1.14 (1.09, 1.19)	**<** **0.001**	3.41 ± 4.70	1.15 (1.09, 1.20)	**<** **0.001**

*Note:* Bold *p* values represent < 0.05.

Abbreviations: BMI = body mass index; CI = confidence interval; CST-30 = 30 s chair stand test; CVD = cardiovascular disease; HbA1c = hemoglobin A1c; DR = diabetic retinopathy; OR = odds ratio.

**Table 3:** Association between muscle strength and DR in different models by gender.

Exposure	Nonadjusted model		Model 1		Model 2		Model 3	
	OR (95% CI)	*p* value	OR (95% CI)	*p* value	OR (95% CI)	*p* value	OR (95% CI)	*p* value
Women								
Low grip strength^a^								
No	Reference		Reference		Reference		Reference	
Yes	0.82 (0.39, 1.72)	0.595	0.89 (0.42, 1.89)	0.753	1.01 (0.42, 2.45)	0.980	0.94 (0.38, 2.29)	0.888
Low CST-30^b^								
No	Reference		Reference		Reference		Reference	
Yes	1.66 (0.98, 2.79)	0.057	1.86 (1.08, 3.19)	**0.025**	2.25 (1.18, 4.32)	**0.014**	2.26 (1.18, 4.34)	**0.014**
Men								
Low grip strength^a^								
No	Reference		Reference		Reference		Reference	
Yes	2.63 (1.34, 5.16)	**0.005**	2.81 (1.40, 5.63)	**0.004**	2.96 (1.32, 6.64)	**0.009**	2.98 (1.33, 6.68)	**0.008**
Low CST-30^b^								
No	Reference		Reference		Reference		Reference	
Yes	1.01 (0.55, 1.86)	0.973	1.01 (0.53, 1.92)	0.970	0.82 (0.40, 1.71)	0.601	0.79 (0.38, 1.67)	0.541

*Note:* Bold *p* values represent < 0.05. Model 1 was adjusted for age and ethnicity. Model 2 was adjusted for Model 1 plus occupation, annual household income, smoking status, CVD, hypertension, hyperlipidemia, BMI, HbA1c, and duration of diabetes. Model 3 was adjusted for Model 2 plus low CST-30 or low grip strength.

Abbreviations: BMI = body mass index; CI = confidence interval; CST-30 = 30 s chair stand test; CVD = cardiovascular disease; HbA1c = hemoglobin A1c; DR = diabetic retinopathy; OR = odds ratio.

^^^a Low grip strength was defined as < 28 kg for men and < 18 kg for women.

^^^b Low CST-30 was identified as scores falling within the lowest two gender-specific quintile.

## Data Availability

The data that support the findings of this study are available on request from the corresponding author. The data are not publicly available due to privacy or ethical restrictions.

## Supporting Information

Supporting Table 1: Variance inflation factors (VIFs) for covariates in multicollinearity assessment.

Supporting Table 2: Sensitivity analysis on the relationship between muscle strength and any retinopathy (ETDRS level ≥ 14).

Supporting Table 3: Sensitivity analysis on the relationship between muscle strength and DR after multiple imputation.

Supporting Table 4: Associations of muscle strength per unit increase with DR.

Supporting Figure 1: Multivariable-adjusted spline plots illustrating the associations between muscle strength measures and the probability of DR.
