# Supplementary material for: Gender-Specific Associations of Muscle Strength With Diabetic Retinopathy in Middle-Aged and Older Chinese Adults: A Cross-Sectional Study
Source: Int J Endocrinol. 2025 Nov 27;2025:8219457. doi: 10.1155/ije/8219457 (PMC12677995; doi:10.1155/ije/8219457)
Supplement: Supporting Information — Additional supporting information can be found online in the Supporting Information section. [file 8219457.f1.docx]

**Gender-specific associations of muscle strength with diabetic retinopathy in middle-aged and older Chinese adults: a cross-sectional study**

**Supporting Information**

**Supporting Table 1:** Variance inflation factors (VIFs) for covariates in multicollinearity assessment.

**Supporting Table 2:** Sensitivity analysis on the relationship between muscle strength and any retinopathy (ETDRS level ≥ 14).

**Supporting Table 3:** Sensitivity analysis on the relationship between muscle strength and DR after multiple imputation.

**Supporting Table 4:** Associations of muscle strength per unit increase with DR.

**Supporting Figure 1:** Multivariable-adjusted spline plots illustrating the associations between muscle strength measures and the probability of DR

**Supporting Table 1.** *Variance inflation factors (VIFs) for covariates in multicollinearity assessment.*

| **Variables** | VIF |
| --- | --- |
| Age | 1.3 |
| Ethnicity | 1.1 |
| Occupation | 1.1 |
| Annual household income | 1.1 |
| Smoking status | 1.6 |
| CVD | 1.1 |
| Hypertension | 1.1 |
| Duration of diabetes | 1.2 |
| HbA1c | 1.1 |
| BMI | 1.1 |

**Abbreviations:**CVD＝Cardiovascular Disease; BMI＝Body Mass Index; HbA1c＝Hemoglobin A1c.

**Note:**All VIF values were below 5, indicating an absence of significant multicollinearity among the covariates.

**Supporting Table 2.** *Sensitivity analysis on the relationship between muscle strength and any retinopathy (ETDRS level ≥ 14).*

| **Exposure** | **Non-adjusted Model** | | **Model 1** | | **Model 2** | | **Model 3** | |
| --- | --- | --- | --- | --- | --- | --- | --- | --- |
|  | **OR (95%CI)** | ***P*-value** | **OR (95%CI)** | ***P*-value** | **OR (95%CI)** | ***P*-value** | **OR (95%CI)** | ***P*-value** |
| **Women** |  |  |  |  |  |  |  |  |
| Low grip strength ^a^ |  |  |  |  |  |  |  |  |
| No | Reference |  | Reference |  | Reference |  | Reference |  |
| Yes | 0.86 (0.45, 1.62) | 0.635 | 0.89 (0.47, 1.71) | 0.731 | 0.99 (0.47, 2.08) | 0.980 | 0.93 (0.44, 1.97) | 0.856 |
| Low CST-30 ^b^ |  |  |  |  |  |  |  |  |
| No | Reference |  | Reference |  | Reference |  | Reference |  |
| Yes | 1.54 (0.97, 2.42) | 0.065 | 1.65 (1.03, 2.65) | **0.038** | 1.89 (1.09, 3.28) | **0.024** | 1.89 (1.09, 3.29) | **0.024** |
| **Men** |  |  |  |  |  |  |  |  |
| Low grip strength ^a^ |  |  |  |  |  |  |  |  |
| No | Reference |  | Reference |  | Reference |  | Reference |  |
| Yes | 2.31 (1.23, 4.37) | **0.010** | 2.45 (1.28, 4.71) | **0.007** | 2.55 (1.20, 5.44) | **0.015** | 2.55 (1.20, 5.44) | **0.015** |
| Low CST-30 ^b^ |  |  |  |  |  |  |  |  |
| No | Reference |  | Reference |  | Reference |  | Reference |  |
| Yes | 1.23 (0.71, 2.13) | 0.453 | 1.28 (0.72, 2.28) | 0.394 | 1.13 (0.59, 2.16) | 0.718 | 1.12 (0.58, 2.16) | 0.745 |

**Bold *P-*values represent＜0.05.**

*^a^ Low grip strength was defined as**＜28 kg for men and＜18 kg for women.*

*^b^ Low CST-30 was identified as scores falling within the lowest two gender-specific quintiles.*

*Model 1 was adjusted for* *age and ethnicity.*

*Model 2 was adjusted for Model 1 plus occupation, annual household income, smoking status, CVD, hypertension, hyperlipidemia, BMI, HbA1c, and duration of diabetes.*

*Model 3 was adjusted for Model 2 plus low CST-30 or low grip strength.*

**Abbreviations:** CI＝Confidence Interval; OR＝Odds Ratio; CVD＝Cardiovascular Disease; BMI＝Body Mass Index; HbA1c＝Hemoglobin A1c; CST-30＝30-Second Chair Stand Test; DR＝Diabetic Retinopathy.

**Supporting Table 3.** *Sensitivity analysis on the relationship between muscle strength and DR after multiple imputation.*

| **Exposure** | **Non-adjusted Model** | | **Model 1** | | **Model 2** | | **Model 3** | |
| --- | --- | --- | --- | --- | --- | --- | --- | --- |
|  | **OR (95%CI)** | ***P*-value** | **OR (95%CI)** | ***P*-value** | **OR (95%CI)** | ***P*-value** | **OR (95%CI)** | ***P*-value** |
| **Women** |  |  |  |  |  |  |  |  |
| Low grip strength ^a^ |  |  |  |  |  |  |  |  |
| No | Reference |  | Reference |  | Reference |  | Reference |  |
| Yes | 0.82 (0.39, 1.72) | 0.595 | 0.89 (0.42, 1.89) | 0.753 | 0.93 (0.38, 2.25) | 0.869 | 0.86 (0.35, 2.11) | 0.741 |
| Low CST-30 ^b^ |  |  |  |  |  |  |  |  |
| No | Reference |  | Reference |  | Reference |  | Reference |  |
| Yes | 1.66 (0.98, 2.79) | 0.057 | 1.86 (1.08, 3.19) | **0.025** | 2.44 (1.28, 4.65) | **0.007** | 2.46 (1.29, 4.69) | **0.006** |
| **Men** |  |  |  |  |  |  |  |  |
| Low grip strength ^a^ |  |  |  |  |  |  |  |  |
| No | Reference |  | Reference |  | Reference |  | Reference |  |
| Yes | 2.63 (1.34, 5.16) | **0.005** | 2.81 (1.40, 5.63) | **0.004** | 3.14 (1.41, 6.97) | **0.005** | 3.16 (1.42, 7.01) | **0.005** |
| Low CST-30 ^b^ |  |  |  |  |  |  |  |  |
| No | Reference |  | Reference |  | Reference |  | Reference |  |
| Yes | 1.01 (0.55, 1.86) | 0.973 | 1.01 (0.53, 1.92) | 0.970 | 0.82 (0.39, 1.71) | 0.601 | 0.79 (0.38, 1.66) | 0.537 |

**Note:** The notes and abbreviations provided in Supporting Table 2 apply equally to Supporting Table 3.

**Supporting Table 4.** *Associations of muscle strength per unit increase with DR.*

| **Exposure** | **Non-adjusted model** | | **Model 1** | | **Model 2** | | **Model 3** | |
| --- | --- | --- | --- | --- | --- | --- | --- | --- |
|  | **OR (95%CI)** | ***P*-value** | **OR (95%CI)** | ***P*-value** | **OR (95%CI)** | ***P*-value** | **OR (95%CI)** | ***P*-value** |
| **Women** |  |  |  |  |  |  |  |  |
| Grip strength (per 1 kg) | 0.99 (0.94, 1.05) | 0.836 | 0.98 (0.93, 1.04) | 0.516 | 0.99 (0.92, 1.06) | 0.729 | 1.00 (0.93, 1.07) | 0.971 |
| CST-30 (per 1 score) | 0.97 (0.92, 1.02) | 0.283 | 0.96 (0.91, 1.02) | 0.159 | 0.95 (0.88, 1.02) | 0.126 | 0.95 (0.88, 1.02) | 0.136 |
| **Men** |  |  |  |  |  |  |  |  |
| Grip strength (per 1 kg) | 0.97 (0.93, 1.01) | 0.113 | 0.96 (0.92, 1.01) | 0.094 | 0.97 (0.92, 1.02) | 0.272 | 0.97 (0.92, 1.03) | 0.303 |
| CST-30 (per 1 score) | 0.99 (0.94, 1.05) | 0.746 | 0.99 (0.93, 1.05) | 0.726 | 1.03 (0.96, 1.10) | 0.399 | 1.04 (0.97, 1.11) | 0.317 |

*Model 1 was adjusted for age and ethnicity.*

*Model 2 was adjusted for Model 1 plus occupation, annual household income, smoking, CVD, hypertension, hyperlipidemia, BMI, HbA1c, and duration of diabetes.*

*Model 3 was adjusted for Model 2 plus mutual adjustment for the other muscle strength continuous variable.*

**Abbreviations:** CI＝Confidence Interval; OR＝Odds Ratio; CST-30＝30-Second Chair Stand Test; CVD＝Cardiovascular Disease.


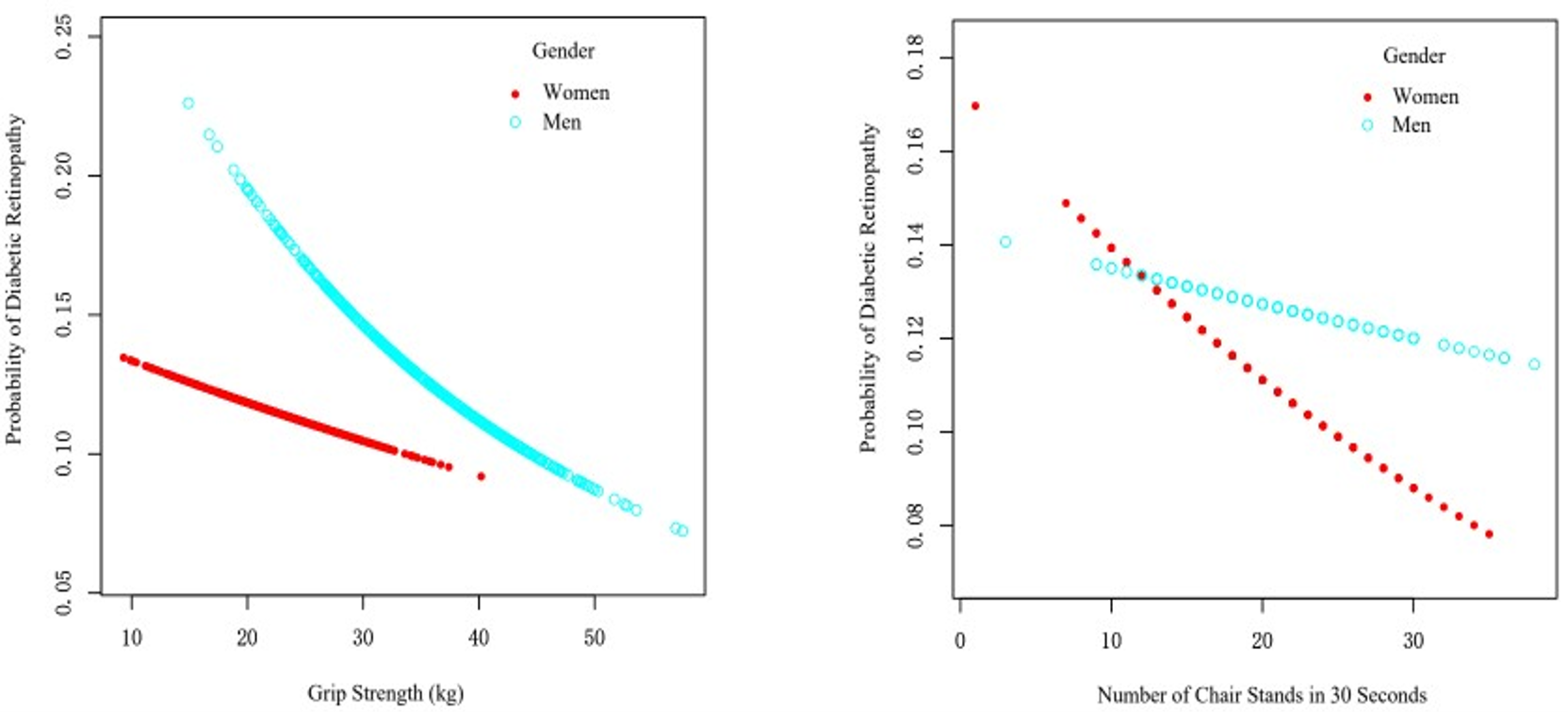


（a） (b)

**Supporting Figure 1.** Multivariable-adjusted spline plots illustrating the associations between muscle strength measures and the probability of DR. Panel (a) shows the relationship between grip strength (kg) and DR; Panel (b) shows the association between the number of chair stands in 30 seconds (CST-30) and DR. Both panels were fitted with spline models adjusted for age, ethnicity, occupation, annual household income, smoking status, cardiovascular disease (CVD), hypertension, hyperlipidemia, body mass index (BMI), glycated hemoglobin (HbA1c), and diabetes duration. Colors denote gender (red: women; blue: men). Red dots and red solid lines represent Women; blue open circles and blue lines represent Men. The nonlinearity terms were not statistically significant (all *P*＞0.2).
